# Multiple Sclerosis and Catastrophic Health Expenditure in Iran

**DOI:** 10.5539/gjhs.v8n9p194

**Published:** 2016-01-22

**Authors:** Yaser Juyani, Dorsa Hamedi, Seyede Sedighe Hosseini Jebeli, Maryam Qasham

**Affiliations:** 1 Health Management and Economics Research Centre, Iran University of Medical Sciences, Tehran, Iran; 2 Musculoskeletal Rehabilitation Research Centre, Department of occupational therapy, Rehabilitation School, Ahvaz Judishapur University of Medical Science, Ahvaz, Iran; 3 Health Services Management Department, School of Public Health, Ahvaz Jundishapur University of Medical Sciences, Ahvaz, Iran; 4 Financial Resource Planning Management, Vice Chancellor of Management Development Resource Planning, Tehran University of medical science, Tehran, Iran

**Keywords:** multiple sclerosis, catastrophic health expenditure, health economics, health insurance

## Abstract

**Background:**

There are many disabling medical conditions which can result in catastrophic health expenditure. Multiple Sclerosis is one of the most costly medical conditions through the world which encounter families to the catastrophic health expenditures. This study aims to investigate on what extent Multiple sclerosis patients face catastrophic costs.

**Method:**

This study was carried out in Ahvaz, Iran (2014). The study population included households that at least one of their members suffers from MS. To analyze data, Logit regression model was employed by using the default software STATA12.

**Results:**

3.37% of families were encountered with catastrophic costs. Important variables including brand of drug, housing, income and health insurance were significantly correlated with catastrophic expenditure.

**Conclusions:**

This study suggests that although a small proportion of MS patients met the catastrophic health expenditure, mechanisms that pool risk and cost (e.g. health insurance) are required to protect them and improve financial and access equity in health care.

## 1. Introduction

Catastrophic health expenditure (CHE) is considered as the spending on health that exceeds a pre-defined percentage of a household’s capacity to pay (Wagstaff & Doorslaer, 2003). The literature indicates different calculations and cutoff points to estimate CHE (Xu, 2003). It has great reflection on people’s lives, discouraging them at times from using healthcare services. Furthermore, this may lead to a reduction in consumption of other essential goods and services, thus exposing families to poverty risk and most of the occasions to economic ruin (Boing, 2014).

Iran is one of the countries which Out-of-pocket payment is a major method for health system financing. This may result in catastrophic health expenditures. In the fifth development plan, protection of households against healthcare costs is clearly emphasized in the form of two goals: 1- Reduction of out of pocket payment to 30%. 2- Reduction in the number of people facing catastrophic health costs to 1%.

A study shows that catastrophic health expenditure among Brazilian families varied between 0.7% and 21.0%, depending on the calculation method. Research findings show socioeconomic inequalities in the CHE between 2002-2003 and 2008-2009 rose significantly, became 5.20 and 4.17 times higher among the poorest and the least educated respectively (Boing, 2014). In China research findings show CHE incidence and intensity were relatively high among elderlies who suffer from chronic conditions. Healthcare insurance did not significantly affect CHE risk (Wang, Li, & Chen, 2015).

In Kenya research findings indicate that the proportion of families facing CHE varies widely between 1.52% and 28.38% depending on the calculation methods. The number of working adults in families and membership in social safety nets appears to decline the risk of catastrophic expenditure. In contrast, seeking care in public or private hospitals increased the risk of CHE (Buigut, Ettarh, & Amendah, 2015). To determine the incidence of catastrophic health expenditure in Nepal, overall 284 of the 1997 households were studied in Kathmandu, 13.8% of them reported catastrophic health expenditure. After adjusting for confounders, this expenditure was found to be associated with injuries; particularly those resulting from road traffic accidents (Saito, 2014). A study explored the burden of out-of-pocket health expenditures among the Korean chronic patients. Roughly 3.5% of the participants experienced CHE. According to the different conditions, households with a member who suffered from cerebrovascular disease, diabetes, or chronic kidney disease were at a significantly higher risk of experiencing CHE (J., Choi, J.-W., Choi, Kim, Yoo, & Park, 2015).

In Iran the proportion of households facing catastrophic healthcare reduced from 12.6% in 2003 to 11.8% in 2008, but this change was not statistically significant. The key determinants of catastrophic healthcare expenditures for both years were healthcare utilization (especially inpatient and dentistry services), economic status, and disabled or elderly family member influenced exposure to catastrophic healthcare expenditure in 2008 (Kavosi, 2009).

In another research which has been done in Iran, more than 3 percent of the households were facing catastrophic health expenditure. Moreover 1.5 percent of the households have been impoverished (Yousefi, Assari Arani, Sahabi, Kazemnejad, & Fazaeli, 2015). In a study among Iranian training hospitals, findings show that 8 variables including gender of the household’s head, health status of the member of household, the size of household, residency in Tehran city, number of previous hospitalization, having a house, the level of income and finally complementary health insurance coverage, were influential factors on exposure to catastrophic medical payments (Ghiasvand, Hadian, Maleki, & Shabaninejad, 2010). In another study it was found that 8.3 Present of the families of Yazd province in Iran were exposed to catastrophic health expenditure. The use of inpatient services has the largest proportion of out-of-pocket payment and its relationship to incidence of catastrophic health expenditure was found to be significant. Moreover it was found that the relationships between family sizes, member ≤ 5 years old, and the use of medical services and catastrophic health expenditure were significant (Amery, Jafari, & Panahi, 2013).

Although numerous studies explored catastrophic health expenditures (CHE) worldwide, most of them focused on whole population rather than specific vulnerable groups. Since families who suffer from certain costly diseases (e.g. Cancer, Multiple Sclerosis) are more likely to face catastrophic health expenditures and Multiple Sclerosis is one of the most costly medical conditions through the world which encounter families to the catastrophic health expenditures, this study analyzed the extent and associated factors of CHE in household with Multiple Sclerosis patients.

## 2. Method

### 2.1 Catastrophic Expenditure

Generally, two thresholds are widely used to define CHE: 1) out-of-pocket healthcare payments (OOP) that comprise ≥10% of total household expenditures (O’Donnell, 2005) and 2) out-of pocket healthcare payments that comprise ≥40% of nonfood household expenditures (Xu, 2003). By deducting food expenses, the latter indicator can partly avoid measurement deviation that poor households which cannot afford to meet catastrophic payments are ignored (Wagstaff & Doorslaer, 2003).

According to the World Health Organization when families spend more than 40% of the ability to pay for cost of health services they face catastrophic health expenditure (Ekman, 2007). We considered WHO threshold (40%) to calculate CHE. So the ratio of household spending for health on ability to pay was calculated. Ability to pay is considered as the entire costs of the household during a given period minus the costs spent on food. We considered all cost of treatment (including drug, hospitalization, doctor visit, rehabilitation services) paid directly by households for MS patients through 1 year from 2014-2015.

#### 2.2 Sample Size

Since there were no relevant studies about the catastrophic cost of Multiple sclerosis, to estimate the sample size we consider P equal to 0.3 regarding to other relevant studies and used bellow formula:


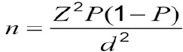


According to the following values, sample size equaled to N=322.

P= 0.3; Z=1.96; d=0.05; N=322.

#### 2.3 Regression Analysis

Researchers usually consider that the dependent variable is continuous set of values. However; there are numerous cases in which the dependent variable has only two values, zero and one. For instance, a person could buy drugs or to unsubscribe. Models which are used for such purposes are called models with qualitative dependent variables (e.g Logit and Probit). Simplest of these models are models in which the dependent variables are binary. In the following function Xi is a binary variable:


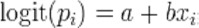


If the non-visible part of the Gumbel distribution is assumed, the difference between the logistic distributions gives the binary Logit model (Long, 1997).


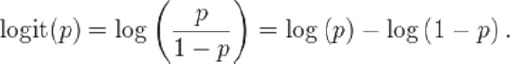


We employed regression analysis in order to find the relationship between CHE and socioeconomic variables in this study. Since facing catastrophic costs (as constant variable), is a binary response, using a binary model was applicable here; so the Logit regression model has been applied in this study.

### 3. Results

#### 3.1 Descriptive Statistics

Descriptive statistics of this study are shown in Table 1. 90% of household`s heads were man and 10% were woman. In current study, 67% of families have their own house and 33% were renter. 68.54% of the drugs which used by participants has been made in Iran and the remaining proportion were imported. In this study, 53% of studied sample had basic health insurance coverage and 47% of them didn’t have any kind of insurance. 3.37% of the families who participated in this study face catastrophic health cost.

**Table 1:** Demographic characteristics of the studied sample (n=322)

Variables		Frequency (%)
**Gender**	Female	9.55
	Male	90.45
	Total	100
**House ownership**	Land lord	66.85
	Renter	33.15
	Total	100
**Drug**	Iranian	68.54
	Foreign	31.46
	Total	100
**Having Basic Health Insurance Coverage**	Yes	52.81
	No	47.19
	Total	100
**Catastrophic Cost**	Yes	3.37
	No	96.63
	Total	100
**Education**	Elementary	6.74
	Secondary	29.21
	Graduated	64.05
	total	100
**Family size**	Mean	4.83
	Maximum	10
	Minimum	2
	Standard deviation	1.37
**Age of household head**	Mean	53.87
	Maximum	76
	Minimum	28
	Standard deviation	8.913
**Income (Rial)**	Mean	19.129.213
	Maximum	75.000.000
	Minimum	1.400.000
	Standard deviation	10.621.971

#### 3.2 Econometric Findings

As shown in Table 2, All independent variables had significant correlation with catastrophic cost (p<0.05) except the severity of Multiple Sclerosis which didn’t show significant correlation with catastrophic cost (p>0.05).

**Table 2:** The associations between the having catastrophe cost and the studied sample’s demographic characteristics

Variables	Coefficients	P-value
**Disease stage**	0.757	0.429
**Drug**	-2.358	0.030
**Family size**	3.278	0.001
**House ownership**	6.486	0.014
**Income**	-1.29	0.023
**Having Basic Health Insurance Coverage**	10.431	0.033
**Sex**	-16.118	0.012
**Number of visits**	0.806	0.001
**Age**	- 0.309	0.002

In logistic regression, the coefficient of R2 is not a good indicator for fitted model, so other kind of indicators is used. In Logit regression, the Mc Fadden R-squared is similar to the R2 statistics in linear regression, and the value varies between 0-1. When the value is closer to 1, the reality adjustment of the model is high. The calculated value in current study is 0.680, (table 3) which is an acceptable value in Logit regression based on previous studies.

**Table 3:** Fitting model indicators

Indicator	Quantity	p-value
LR statistic	107.202	0.000≥
McFadden R-squared	0.680	-------
Log likelihood	-25.112	-------

In order to test the hypothesis that independent variables have no effect on dependent variables, the LR statistics is used. The hypothesis that all coefficients are equal to zero (0) in contrast to none of them is tested. The P-value is equal to 0, so it is less than 0.05, and this means that the H0 hypothesis is not acceptable and the regression model is statistically significant. Log likelihood shows the fitness of model and its value is always negative. The calculated value in this study is acceptable based on similar researches.

## 4. Discussion

Among all variables which have been studied, only disease stage had no significant correlation with exposure to catastrophic costs. Drug type had significant negative correlation (p= 0.03) with catastrophic cost which means that by using Iranian drug type, the catastrophic cost would decrease. Considering the higher cost of foreign drugs, this result seems reasonable.

The family size had a significant positive correlation (p<0.001) with catastrophic cost, which means that by increasing the family size, the catastrophe cost will increase. This finding is compatible with previous studies in Iran (Ghiasvand, 2010; Amery, 2013).

Owning a house had a positive significant relationship with catastrophe cost. It means that the patients with their own house are more likely to interface with catastrophic cost in comparison with the renters. This result seems unreasonable and should be more investigated. Family income had significant negative correlation (P=0.02) with catastrophic cost. It is clear that when household`s income increases, probability of facing catastrophic cost could decrease.

The results indicated a significant positive relationship (p=0.03) between public health insurance coverage and catastrophic cost, which means that families who had public health insurance coverage, had more catastrophe cost. This result is somehow different with the results of other studies. In China there were no significant relationship between health insurance and exposure to CHE (Wang, Li, & Chen, 2015). While in Kenya; membership in social safety nets reduced the risk of facing CHE (Buigut, 2015). In another study in Iran, basic health insurance coverage had no significant effect on reducing exposure to CHE (Ghiasvand, 2010). In the study of factors affecting specialty drug`s demand in Iran, there were no significant relationship between health insurance and demand of drug (Jebeli, Barouni, Orojloo, & Mehraban, 2014).

In this research, the cause of these results maybe is due to the adverse selection of insurance coverage where the healthy and younger families are less likely to join insurance funds, so the covered families are older and suffer poor health condition. The other factor that may effect on this result is the moral hazard of insured people and physicians. Although it does not seem reasonable that people with public health insurance coverage continue the use of services till they encounter to catastrophe cost, but this may occur by physician induced demand.

A Significant negative relationship was seen between gender and catastrophic cost. It means that families with man household`s head are less likely to face CHE. It seems reasonable as men are more able to be employed and earn income.

The number of physician’s visits had a significant positive relationship with catastrophe cost, obviously by increasing the number of visits, the treatment cost would increase and the probability of the catastrophe cost would rise.

The age of household`s head had a significant negative correlation with the probability of facing catastrophic cost, which suggests that the younger people who have lower income and fund, have a higher risk of encountering catastrophic cost.

## 5. Conclusion

Since this study has been done after the implementation of a national financial safety program in Iran (2014), the rate of CHE was far less than what we expected. So we conclude that this program which was funded by ministry of health has been successful in reducing the risk of facing CHE among vulnerable groups such as Multiple Sclerosis patients.
